# Antimicrobial Activity of Some Water Plants from the Northeastern Anatolian Region of Turkey

**DOI:** 10.3390/molecules14010321

**Published:** 2009-01-12

**Authors:** Hanife Özbay, Ahmet Alim

**Affiliations:** 1 Kafkas University, Faculty of Science, Department of Biology 36100 Kars, Turkey; 2 Public Health Laboratory 58100 Sivas, Turkey

**Keywords:** Antimicrobial activity, *Butomus umbellatus*, *Polygonum amphibium* L. (*Persicaria amphibian* L.), *Sparganium emersum*, *Sparganium erectum*.

## Abstract

The antimicrobial activity of methanol and acetone extracts of *Butomus umbellatus, Polygonum amphibium,* and two species of the genus *Sparganium* (*S. erectum* and *S. emersum*) against three Gram-positive, five Gram-negative bacteria and one fungus was assessed by the disk diffusion method. The microorganisms used were *Staphylococcus aureus* ATCC-29740**,** *Escherichia coli* ATCC-25922, *Pseudomonas aeruginosa* ATCC-15442, *Salmonella typhi* NCTC-9394, *Klebsiella pneumoniae* NCTC-5046, *Proteus vulgaris* ATCC-7829, *Bacillus subtilis* ATCC-6633, *Corynebacterium diphteriae* RSHM-633 and *Candida albicans* ATCC-10231. Methanol extracts of the plants did not exhibit any inhibitory activity against any of the microorganisms, while the acetone extracts of the all tested plants only showed significant activity against *Bacillus subtilis*, with inhibition zones and minimal inhibitory concentration values in the 7-16 mm and 0.49-12.50 mg/mL ranges, respectively.

## Introduction

Interest in plant-derived drugs has been increasing, mainly due to the current widespread belief that “green medicine” is safer and more dependable than costly synthetic drugs, many of which have adverse side effects [1].

The monotypic genus *Butomus* is the only member of the *Butomaceae* family, which is allied to *Alismataceae* and the non-European *Limnocharitaceae* [2]. The genus is widespread in the flora of Turkey and is represented by one species [3]. *B. umbellatus* L. is known as *su meneksesi* in Turkey and grows as a submerged or emergent aquatic or as a terrestrial species near the water’s edge. It has edible tubers that contain more than 50% starch. The seed is also edible [4,5,6]. Literature describing the chemical and antimicrobial activity of the genus *Butomus* is not available at present.

The genus *Polygonum* (*Polygonaceae*) consists of about 300 species, which are distributed worldwide, mostly in the Northern temperate region [7]. The diversity of the genus *Polygonum*, which comprises 25 species, is not well known in the aquatic habitats of Turkey, but it has been recorded in the eastern part of the country [8]. *P. amphibium* is known as *su cobandegnegi* in Turkey. As an aquatic, *P. amphibium* L. grows in standing or slowly moving water in lakes, reservoirs, canals, ditches, large fenland drains and sluggish streams and rivers [2].

In folk medicine and culture, *P. amphibium* has been valued for its tannin content, as a diuretic, as a cheap substitute for sarsaparilla in the USA [9], and as a source of yellow dye in the Shetland and Fair Isles [10]. The whole plant, especially the root, has an astringent, depurative skin [11,12]. An infusion of the leaves and stems is used to treat stomach pains and children with diarrhoea [13]. The root can be eaten raw, and an infusion of the dried, pounded roots is used in the treatment of chest colds [13]. A poultice of the fresh roots is applied directly to the mouth to treat blisters [13]. Compounds such as flavonoids [14,15,16,17], triterponoids [18], anthraquinones [19,20], coumarins [21], phenylpropanoids [22,23], lignans [24], sesquiterponoids [25], stilbenoids [26] and tannins [7] were identified in several species of *Polygonum*. Although chemical data is available at the generic level, information on the microbial properties or chemical composition of *P. amphibium* is not available in the literature.

There are 14 species in the genus *Sparganium,* all of them aquatic perennial herbs [2]. Three species have been identified in Turkey [27]. *S. erectum* L. is known as *dik sıgırsazı* in Turkey. Another *Sparganium* species, *S. emersem* Rehmann, is known in Turkey as *yalın sıgırsazı*. In folk medicine an infusion of whole *S. erectum* mixed with other plant leaves has been used in the treatment of chills [13]. Some species belonging to the genus *Sparganium* contain a variety of metabolites. including flavonoids, essential oil, phenylpropanoid glycosides and aromatic alkenes [28]. No literature about the antimicrobial activity of the genus *Sparganium* is available.

All submersed aquatic angiosperms are secondarily adapted for life in water. Unlike their terrestrial counterparts, where water is a persistent feature only in the subsoil environment, submersed aquatic angiosperms are surrounded by water. Submersed aquatic angiosperms have attracted the interest of researchers because they show promise as antimicrobial agents [29, 30].

Although the antimicrobial activities and other properties of numerous terrestrial angiosperms have received a great deal of attention, perhaps because of their extensive occurrence, so far, aquatic angiosperms from rivers, lakes etc. have received less attention. In this preliminary antimicrobial assay, we now wish to report on the antimicrobial activity of the methanol and acetone extracts of the aquatic plants *P. amphibium, B. umbellatus, S. emersum*, and *S. erectum.*

## Results and Discussion

The inhibitory activities of the extracts of *S*. *erectum* and *S. emersum, B. umbellatus* and *P. amphibium* are given in Table 1. Acetone extracts of the whole plant of *S*. *erectum*, *S. emersum, P. amphibium* and *B*. *umbellatus* were inactive against *Staphylococcus aureus***,** *Escherichia coli, Pseudomonas aeruginosa,* *Salmonella typhi,* *Proteus vulgaris* and *Corynebacterium diphteriae* bacterial species, as well as a fungal species. However, acetone extracts of all tested plants were active against *B. subtilis*. Inhibition zones of *S*. *erectum,* *B*. *umbellatus*, *S. emersum* and *P. amphibium* for *B. subtilis* were 16, 12, 7 and 12 mm respectively. The highest inhibition zone was produced by the extract of *S*. *emersum.* MIC’s of the plant extracts for *B. subtilis* were 0.49 mg/mL (*S. erectum),* 2.50 mg/mL (*B*. *umbellatus)*, 12.50 mg/mL (*S. emersum)* and 2.30 mg/mL (*P. amphibium).* The lowest MIC was observed with the extract from *S. erectum.* On the other hand no inhibitory activities have been determined with the methanol extracts of the plants against any of the microorganisms tested (data not shown).

**Table 1:** Results of the antimicrobial activities of the whole plant extract of *S. erectum*, *B. umbellatus, S. emersum,* and *P. amphibium.*

^a^DD						
Microorganizms	*S. erectum*	*B. umbellatus*	*S. emersum*	*P. amphibium*	Gen^b^	Nys^c^
						
*Staphylococcus aureus*	-	-	-	-	23±0.76	-
*Escherichia coli*	-	-	-	-	16±0.96	-
*Pseudomonas aeruginosa*	-	-	-	-	20±1.06	-
*Salmonella typhi*	-	-	-	-	10±0.45	-
*Klebsiella pneumonia*	-	-	-	-	20±0.70	-
*Proteus vulgaris*	-	-	-	-	22±1.40	-
*Bacillus subtilis*	16±0.8	12±1.4	7±1.4	12±0.8	29±1.15	-
*Corynebacterium diphteriae*	-	-	-	-	23±1.10	-
*Candida albicans*	-	-	-	-	-	25±0.90
^MIC (mg/mL)^						
*Bacillus subtilis*	0.49	2.50	12.50	2.30		

^a^DD, agar disc diffusion method. Diameter of inhibition zone (mm) including disk diameter of 6 mm.

^b^Gentamycin (antibacterial).

^c^Nystatin (antifungal).

According to MIC values, *S. erectum* extracts showed moderate activity against *B.* *subtilis* while the other three extracts were inactive [31]. All tested plant extracts only inhibited the growth of *B. subtilis*. Although they may contain common chemical constituents [28] two species of *Sparganium* belonging to the *Sparganiaceae* family revealed different inhibition zones and MIC’s for *B. subtilis*. These differences could be due to their different habitats, as *S. erectum* grows at the edge of waters as an emergent species while *S. emersum* grows in water with 0.2-1 m deep as a submersed plant. Because submersed aquatic angiosperms are secondarily adapted to life in water, their antimicrobial activity may possible increase with the increasing terrestrial life contact. 

In contrast to our findings on *P. amphibium,* numerous authors have reported various degrees of antibacterial and antifungal activity of the various crude extracts obtained from the genus *Polygonum* belonging to the *Polygonaceae* family. In an earlier antifungal assay, Mohamed *et al*. [32] examined the antimycotic activity of an 80% ethanol extract of *P. minus* against seven fungal species and they reported inhibitory activity only against *A. alternate*. In another antifungal screen, dichloromethane and methanol extracts of *P. hydropiperoides* were tested against eleven fungal species. Unlike the methanol extract, the dichloromethane extract was effective against *C*. *albicans,* *C. cladosporioides,* *C. neoformans*, and *M. gypseum* [33]. On the other hand inhibitory activities of dichloromethane, methanol, 50% of ethanol and aqueous extracts of *P. punctatum* were tested against *B*. *subtilis*, *M*. *luteus*, *S. aureus*, *E. coli*, *P. aeruginosa*, *C*. *albicans*, *Mucor* spp., and *A*. *niger* [34] and the methanol extract only showed activity against *B*. *subtilis.* Our study, in which we did not observe any antimicrobial activity with methanol extracts, appears to match this finding. Similarly in a recent study ether, ethanol and hot water extracts of *P. cognatum* were tested against *S*. *aureus*, *B*. *subtilis*, *E*. *coli*, *P*. *aeruginosa* and *C*. *albicans.* Inhibitory effects of the ether and ethanol extracts were found against *S*. *aureus* and *B*. *subtilis* [35]. The extraction procedure, solvent type and species differences might be the reason for the differences in antimicrobial effects against the test organisms as exhibited by the genus *Polygonum*. 

Unlike the acetone one, the methanol extract revealed no significant inhibitory activity against any of the microorganisms and we may conclude that the active compounds in our test plants reside in acetone extracts rather than methanol extracts, and that acetone seems to be better than methanol for extracting antimicrobial compounds from medicinal plants. Eloff reported similar findings for extracting of antimicrobial compounds from some medicinal plants [36]. He suggested that acetone dissolves many hydrophilic and lipophilic components from the medicinal plants, is miscible with water, is volatile and has a low toxicity to the bioassay used [36]. 

## Conclusions

The present results suggest that there are many unexploited plants and natural compounds in aquatic plant species that could be potential resources for the reduction or control of bacterial diseases. Therefore, further studies should be undertaken to characterise the active compounds residing in these types of plants. Additionally, evaluation of the effects of each individual compound on microorganisms as well as toxicological studies need to be performed.

## Experimental

### Plant material

Collection of all test plants took place between June and July 2006. *S*. *erectum*, *S. emersum* and *B. umbellatus* were collected from Çali Lake (40° 31’N, 43° 15’E) and *P. amphibium* was collected from Aktaş Lake (41° 12’N, 43° 12’E). 

### Preparation of extracts

After collection, all parts of the plant material were cleaned with tap water and then with distilled water. Plants were air dried at ambient temperature and powdered with a Waring blender. Plant material was extracted by using a Soxlet extractor with solvents of increasing polarity beginning with acetone, followed by methanol (Merck, Darmstadt). The solvents used in this work were purified [37] before extraction. Extraction of each plant powder (20 g) was done at room temperature 150 ml solvent. Extraction with each solvent was carried out for 8 to 10 h. 

### Antimicrobial assay

The antimicrobial and antifungal activity of the acetone extract was evaluated against three Gram-positive, five Gram-negative bacteria and one fungus by the disk diffusion method. The microorganisms used were *Staphylococcus aureus* ATCC-29740, *Escherichia coli* ATCC-25922, *Pseudomonas aeruginosa* ATCC-15442, *Salmonella typhi* NCTC-9394, *Klebsiella pneumoniae* NCTC-5046 , *Proteus vulgaris* ATCC-7829, *Bacillus subtilis* ATCC-6633, *Corynebacterium diphteriae* RSHM-633 and *Candida albicans* ATCC-10231. Cultures were obtained from the culture collections of the Department of Health of Refik Saydam Hygiene Centre, Contagious Diseases Research Department (Ankara, Turkey). Bacterial strains were cultured overnight at 37 °C in Mueller Hinton Agar (Oxoid-CM 337). Yeast was cultured overnight at 30 °C in Sabouraud dextrose agar (Oxoid-CM41). All the experiments were carried out in triplicate and the average and standard deviation (SD) were calculated for the inhibition zone diameters.

The agar disc diffusion method was employed for the determination of antimicrobial activities of the extract [38]. Briefly, a suspension of the tested microorganism (0.1 mL 10^8^ cells per mL) was spread on the solid media plates. Filter paper discs (6 mm in diameter) were impregnated with 15 µL of the extract and placed on the inoculated plates. These plates, after staying at 4 °C for 2 h, were incubated at 37 °C for 24 h for bacteria and at 30 °C for 48 h for the yeast. The diameters of the inhibition zones were measured in millimetres. 

A broth microdilution susceptibility assay was used, as recommended by NCCLS, for the determination of MIC [39]. All tests were performed in Mueller Hinton broth (MHB; OXOID-CM405) with the exception of the yeasts (Sabouraud dextrose broth-SDB; DIFCO). Bacterial strains were cultured overnight at 37 °C in MHA (Mueller Hinton Agar) and the yeasts were cultured overnight at 30 °C in SDA (Sabouraud dextrose agar). Test strains were suspended in MHB to give a final density of 5×10^5^ cfu/mL and these were confirmed by viable counts. Geometric dilutions ranging from 1/2 mg/mL to 1/6,400 mg/mL of the extract were prepared in a 96-well microtiter plate, including one growth control and one sterility control. Plates were incubated under normal atmospheric conditions at 37 °C for 24 h for bacteria and at 30 °C for 48 h for the yeasts. The MIC of amikacin, clindamycine and ciprofloxacine was individually determined in parallel experiments in order to control the sensitivity of the test organisms. Bacterial growth was indicated by the presence of a white “pellet” on the well bottom.
